# Coordinated and Cohesive Movement of Two Small Conspecific Fish Induced by Eliciting a Simultaneous Optomotor Response

**DOI:** 10.1371/journal.pone.0011248

**Published:** 2010-06-22

**Authors:** Haruka Imada, Masahito Hoki, Yuji Suehiro, Teruhiro Okuyama, Daisuke Kurabayashi, Atsuko Shimada, Kiyoshi Naruse, Hiroyuki Takeda, Takeo Kubo, Hideaki Takeuchi

**Affiliations:** 1 Department of Biological Sciences, Graduate School of Science, The University of Tokyo, Tokyo, Japan; 2 Department of Mechanical and Control Engineering, Tokyo Institute of Technology, Tokyo, Japan; 3 Laboratory of BioResource, National Institute for Basic Biology, Okazaki, Aichi, Japan; University of Bristol, United Kingdom

## Abstract

**Background:**

In animal groups such as herds, schools, and flocks, a certain distance is maintained between adjacent individuals, allowing them to move as a cohesive unit. Proximate causations of the cohesive and coordinated movement under dynamic conditions, however, have been poorly understood.

**Methodology/Principal Findings:**

We established a novel and simple behavioral assay using pairs of small fish (medaka and dwarf pufferfish) by eliciting a simultaneous optomotor response (OMR). We demonstrated that two homospecific fish began to move cohesively and maintained a distance of 2 to 4 cm between them when an OMR was elicited simultaneously in the fish. The coordinated and cohesive movement was not exhibited under a static condition. During the cohesive movement, the relative position of the two fish was not stable. Furthermore, adult medaka exhibited the cohesive movement but larvae did not, despite the fact that an OMR could be elicited in larvae, indicating that this ability to coordinate movement develops during maturation. The cohesive movement was detected in homospecific pairs irrespective of body-color, sex, or albino mutation, but was not detected between heterospecific pairs, suggesting that coordinated movement is based on a conspecific interaction.

**Conclusions/Significance:**

Our findings demonstrate that coordinated behavior between a pair of animals was elicited by a simultaneous OMR in two small fish. This is the first report to demonstrate induction of a schooling-like movement in a pair of fish by an OMR and to investigate the effect of age, sex, body color, and species on coordination between animals under a dynamic condition.

## Introduction

**Figure 1:**
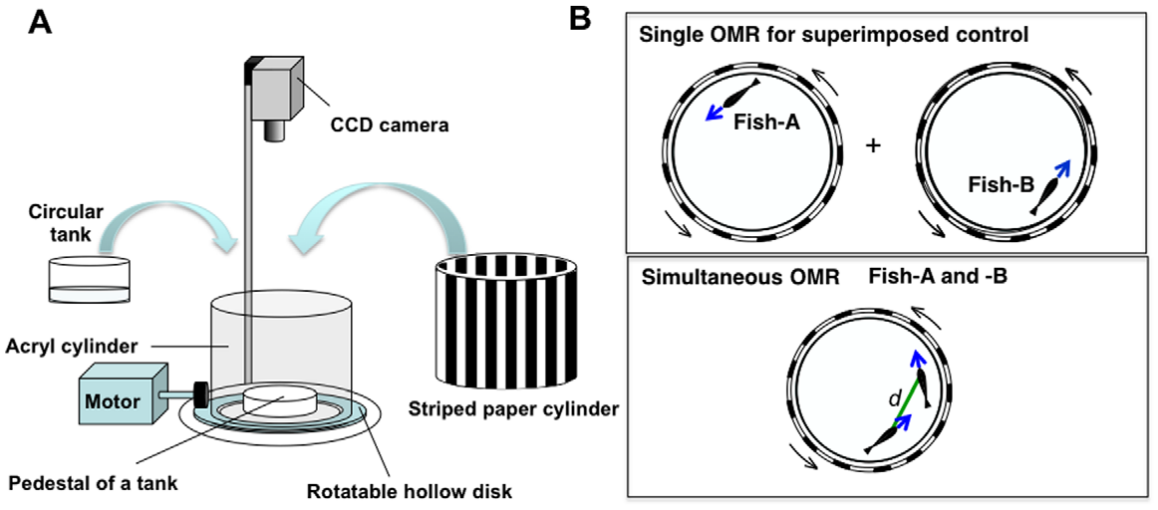
Apparatus and procedure for the OMR. **(A) Apparatus used for measurement of the OMR.** A circular test tank was fixed on the pedestal and did not move. A striped paper cylinder was fixed on the inner surface of the acrylic cylinder. **(B) OMR measurement procedure.** First, the OMR was induced in a single fish to generate the control data for the superimposed condition, to show that the two fish follow the stripe independently (Upper panel). Second, a simultaneous OMR was induced in the two fish. The striped cylinder rotated counter-clockwise (shown by arrows) around the fish tank.

Animal congregations, such as herds [1], flocks[2], and schools [3]–[11], have long fascinated scientists in the field of biology as well as in the fields of engineering and mathematics because of the beauty of their synchronized and coordinated motions. Some types of fish maintain a certain distance between individuals in the same group when the fish group exhibits schooling behavior [3]–[11]. In the 1970s, Partridge and Pitcher reported that when a group of saltwater fish exhibited an optomotor response (OMR) in a large tank (10-m in diameter), schooling behavior was observed [7]. It is generally known that when a striped cylinder rotates, fish move to follow the rotating stripe pattern, exhibiting an OMR to maintain a fixed visual field [12],[13]. To analyze the coordinated movement of individual fish using a simple apparatus, we prepared a small tank (14-cm in diameter) to artificially induce small fish to move by eliciting an OMR under laboratory conditions (Fig. 1A). We used a small laboratory fish, the medaka (*Oryzias latipes*), a freshwater teleost native to East Asia. We used medaka for this experiment for the following reasons. (1) Medaka (*Oryzias latipes*) exhibit prominent shoaling behavior. For example, a single medaka fish tends to approach a conspecific group [14] as well as a mirror image of its own figure [15]. (2) When medakas form a school, individual fish tend to maintain a nearest-neighbor distance [16],[17].

We analyzed interindividual interactions between two fish in a single experiment and demonstrated that coordinated and cohesive behavior (schooling-like behavior) can be induced, even between only two individuals, by eliciting an OMR in two small fish.

## Results

### Two adult medaka with a simultaneous OMR exhibit coordinated and cohesive movement

**Figure 2:**
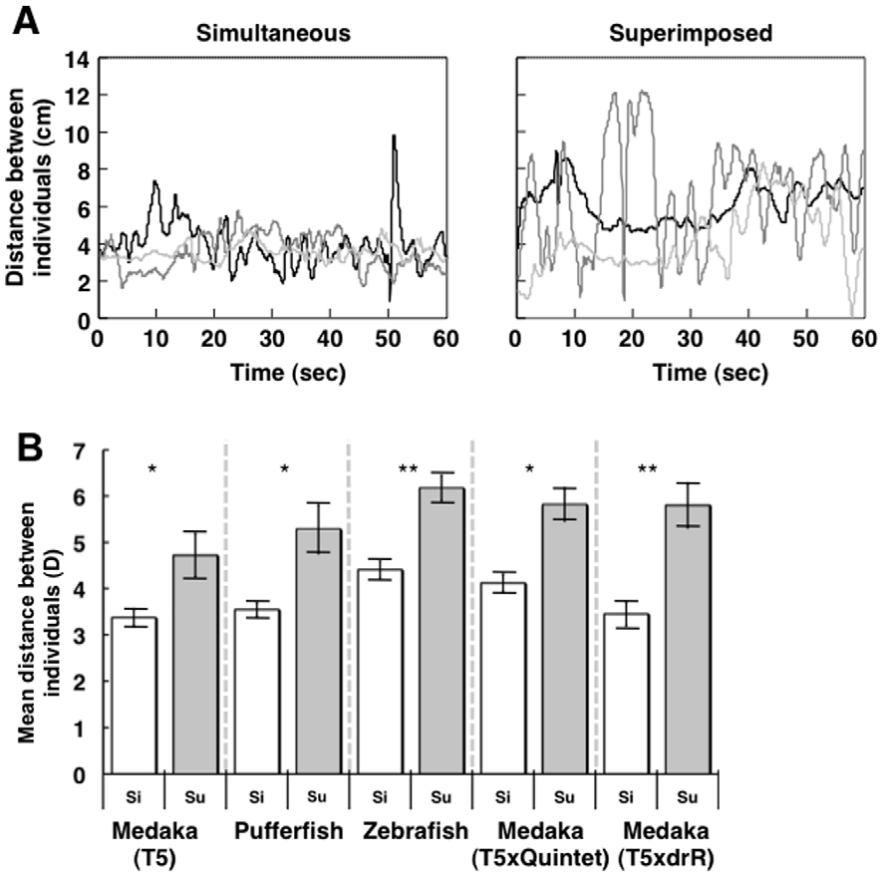
Distance between two adult conspecific fish exhibiting a simultaneous OMR. The rotation speed of the stripes was 60 rpm. (A) Three examples of a temporal change in the distance of the two adult female medaka with a simultaneous OMR (left; ‘simultaneous’). Each line represents one pair of fish. The distance tended to be maintained within 2 to 4 cm. In contrast, the superimposed control data (right;‘superimposed’) ranged widely. (B) The mean distance (D) between two adult female medaka (T5 strain) during elicitation of the OMR (Wilcoxon signed-ranks test: T = 3, N = 10, P = 0.013), adult pufferfish pairs. (Wilcoxon signed-ranks test: T = 4, N = 10, P = 0.017), adult male zebrafish pairs. (Wilcoxon signed-ranks test: T = 2, N = 13, P = 0.002), adult medaka pairs with different body colors (T5 strain and albino mutant [Quintet])) (Wilcoxon signed-ranks test: T = 3, N = 10, P = 0.013), and other adult medaka pairs of different body colors (n = 14) (male T5 and orange-red strains [male drR]). Error bars indicate standard error (SE). **P<0.01, *P<0.05: Wilcoxon signed-ranks test was used for comparison of the difference in the mean distance (D) in each pair between the simultaneous OMR condition (Si) and superimposed control condition (Su).

To examine whether two adult medaka with a simultaneous OMR exhibit schooling-like behavior, we measured the distance between the two fish (d in Fig. 1B) using a video tracking system. The temporal change in distance over 1 min suggested that the two medaka fish swam cohesively and maintained a constant distance apart (Fig. 2A “simultaneous”, Movie S1). There are two possible explanations for this coordinated movement. First, the two fish followed the striped cylinder independently. Second, the two fish followed the striped cylinder in a coordinated manner that is mediated by an interindividual interaction. To test these possibilities, we generated control data that represented movement of the two fish following the stripe independently. First, we recorded the OMR for each single fish, Fish A and B, for 1 min (Fig. 1B). Then, we recorded the OMR simultaneously in both fish, A and B, for 1 min (“simultaneous OMR”; Fig. 1B). We then superimposed the recording of the movement of a single Fish A onto that of a single Fish B to generate the control data (”superimposed control”), based on the assumption that superimposed control represents movement of the two fish when they follow the stripes independently. A strong tendency to maintain a constant distance between the two fish while performing a simultaneous OMR, compared with that in the superimposed control, would indicate that the coordinated movement was mediated by an interindividual interaction.

The distance varied within a small range when a simultaneous OMR was elicited in the fish, whereas in the superimposed control, the distance ranged much more widely (Fig. 2A). Similar tendencies were observed for other pairs (Fig. S1A). In the simultaneous OMR condition, the average coefficient of variation (CV) was 0.21, while that in the superimposed control condition was 0.47. In the simultaneous OMR, the mean distance of each pair (D ± SE) was 3.37±0.18 cm, while that in the superimposition was 4.72±0.47 cm. There was significant difference in parameter D between the two groups; Fig. 2B (Wilcoxon signed-ranks test: T = 3, N = 10, P<0.05). This finding indicated that the tendency to maintain a certain distance was mediated by an interindividual interaction. Furthermore, we tested male-male and male-female pairs and confirmed that the coordinated movement emerged irrespective of sex (Fig. S2). We also tested a faster rotating speed of the striped cylinder (80 rpm) and confirmed similar tendencies (Fig. S2B and C). We also analyzed the distance between the two fish under a static condition and demonstrated that the mean distance between the two fish (D ± SE) was 4.81±1.77 cm (Fig. S3A and B), suggesting that the coordinated movement in a pair of fish was induced by the OMR elicitation. To exclude the possibility that the OMR test affected their ability to follow the striped cylinder, we also performed the simultaneous OMR before the single OMR. The change in the order of the OMR test gave the same results (Fig. S3C).

**Figure 3:**
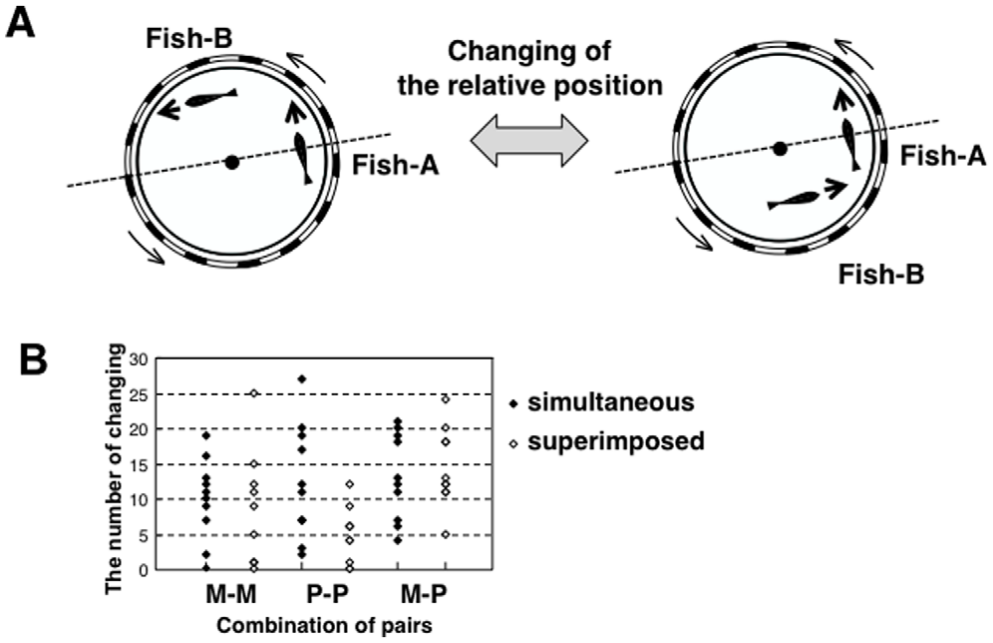
The number of changes in the relative position in each pair. (A) Definition of the two conditions in the relative position in a pair of fish. (B) The number of changes between the two conditions. Three types of pairs were tested (M-M: medaka (T5) pairs, P-P: puffer-puffer pairs, M-P: medaka-puffer pairs). The data used here are identical to those in Fig. 2B (Medaka, and pufferfish) and Fig. 5B (M-P).

We then analyzed the relative positions of the two fish. We defined two conditions based on the relative position of the two fish: (1) Fish B seems to go ahead of Fish A (2) Fish A seems to go ahead of Fish B (Fig. 3A). We counted the number of changes between the two conditions and demonstrated that the number of changes between the two conditions of relative position ranged from 2 to 19 times over 1 min (Fig. 3B M-M). In some cases the two fish changed their relative position frequently (Movie S4). This finding indicated that the relative position of the two fish is not stable during cohesive movement.

### Two larval medaka with a simultaneous OMR did not exhibit coordinated and cohesive movement

**Figure 4:**
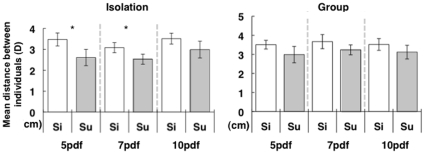
Distance between two medaka larvae exhibiting an OMR. The size of the apparatus for the larval fish was smaller than that for the adult fish. The rotation speed of the stripes was 40 rpm. The mean distance between two larvae reared in isolation for 5 days (left, Wilcoxon signed-ranks test: T = 8, N = 10, P = 0.046), 7 days (middle, Wilcoxon signed-ranks test: T = 8, N = 10, P = 0.046), and 10 days (right, Wilcoxon signed-ranks test: T = 15, N = 10, P = 0.20) after hatching and that between two larvae reared in a group for 5 days (left, Wilcoxon signed-ranks test: T = 8, N = 17, P = 0.28), 7 days (middle, Wilcoxon signed-ranks test: T = 15, N = 10, P = 0.20), and 10 days (right, Wilcoxon signed-ranks test: T = 15, N = 10, P = 0.28) after hatching. Error bars indicate standard error (SE). **P<0.01, *P<0.05: Wilcoxon signed-ranks test was used for comparison of differences in the mean distance for each pair between the simultaneous OMR condition and superimposed control condition. No significant difference was detected between Si and Su except in 5-days or 7-days larvae reared in isolation.

To evaluate whether early larval medaka have the ability to maintain a coordinated distance, we tested larval medaka 5 days, 7 days, and 10 days after hatching. Larval medaka exhibited an OMR activity, although the abilities were relatively poor compared with those of adult medaka. (Table S1 and Movie S5). We also tested group-reared and isolation-reared fish pairs to examine the effect of the environment during development on the behavior. The frequency histogram of the distance distribution did not have a significant peak (Fig. S4) and there was no significant difference of D between the two data sets (simultaneous OMR data or superimposed control data) in any condition except in 5-days or 7-days larvae reared in isolation. (Fig. 4), suggesting that the schooling-like behavior is developed during post-hatch growth.

### Homospecific adult pairs of freshwater pufferfish and zebrafish with a simultaneous OMR exhibit cohesive movement

In the next experiment, we examined whether this tendency is observed in other species. We used a freshwater pufferfish (*Carinotetraodon travancoricus*) called a dwarf puffer, the body length of which is approximately 3 cm, similar to medaka. Although there are no reports on pufferfish shoaling, we demonstrated that a pair of pufferfish maintained 2–4 cm distance between them (Movie S2) and frequency histograms of the simultaneous OMR and superimposed control conditions were significantly different (Fig. 2B and Fig. S1B). In the simultaneous OMR condition, the mean distance (D ± SE) was 3.54±0.15 cm, while that in the superimposed control condition D ± SE was 5.29±0.52 cm. There was a significant difference in the D parameter between the two groups (Wilcoxon signed-ranks test: T = 4, N = 10, P<0.05). The same analysis was performed using zebrafish. The OMR abilities of zebrafish were very low compared with medaka and pufferfish (Table S1). The zebrafish often came to a sudden halt or moved in opposite direction when they were exhibiting an OMR. The zebrafish pairs, however, showed cohesive movement. In the simultaneous OMR condition, the mean distance (D ± SE) was 4.40±0.19 cm, while that in the superimposed control condition were 6.17±0.28 cm. (Fig. 2B and Fig. S1C). There was a significant difference in the D parameter between groups (Wilcoxon signed-ranks test: T = 2, N = 13, P<0.01). We also tested a faster rotating speed (80 rpm) of the striped cylinder and confirmed similar tendencies (Fig. S2D). These findings indicate that coordinated movement in both fish species was mediated by an interindividual interaction.

### Conspecific interaction may mediate the cohesive movement

**Figure 5:**
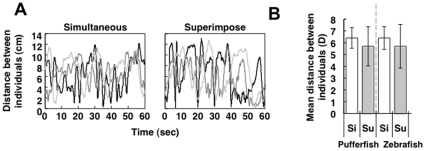
Distance between two adult xenogeneic fish exhibiting a simultaneous OMR. The rotation speed of the stripes was 60 rpm. (A) Three examples of a temporal change in the distance in the simultaneous OMR condition and the superimposed control data between adult medaka and puffer. Each line represents one pair of fish. (B) The mean distance between adult medaka and puffer (Wilcoxon signed-ranks test: T = 19, N = 10, P = 0.21) and that between adult medaka and zebrafish (Wilcoxon signed-ranks test: T = 28, N = 10, P = 0.53). Error bars indicate standard error (SE). **P<0.01, *P<0.05: Wilcoxon signed-ranks test was used for comparison of differences in the mean distance for each pair between the simultaneous OMR condition and superimposed control condition. No significant difference was detected between Si and Su.

To examine whether the cohesive movement is observed in heterospecific combinations, we analyzed pufferfish-medaka and zebrafish-medaka pairs. The distances between the two fish with a simultaneous OMR were not stable (Movie S3 and Fig. 5). The simultaneous OMR and superimposed control histograms of the pufferfish-medaka pairs or zebrafish-medaka pairs did not differ significantly (Fig. S5). There was no significant difference in the distance between the simultaneous and superimposition groups (Fig. 5A and 5B) (Medaka vs pufferfish, Wilcoxon signed-ranks test: T = 19, N = 10, P = 0.21; medaka vs zebrafish, T = 28, N = 10, P = 0.53). These findings strongly suggested that the pufferfish-medaka pairs or the zebrafish-medaka pairs did not show cohesive movement. We also tested a faster rotating speed (80 rpm) of the striped cylinder and confirmed similar tendencies (Fig. S5C). These results suggested that the coordinated movement observed in the three fish species was mediated by a homospecific interaction. To examine the possible importance of body color for a conspecific interaction, we tested hetero-medaka strain pairs with different body colors, using an albino mutant and T5 medaka pairs. Coordinated movement emerged between the pairs using two different hetero-medaka strains that gave the same result (Fig. 2B, Fig. S1D and S1E).

## Discussion

These findings represent the first evidence of the ability of small laboratory-fish pairs to maintain a set distance from other conspecific fish upon the elicitation of a simultaneous OMR. Some properties of the coordinated movement are similar to those observed in fish schools. In schools of cod (*Gadus morhua*), saithe (*Pollachius virens*), and herring (*Clupea harengus*), the nearest neighbor distance (NND) is maintained within a set range, and the relative positions are not stable [4],[6]. In the schooling medaka group (6 individuals) in an aquarium (98 cm square and 10 cm deep), the NND is maintained within 2–3 cm [17], which is consistent with the result using a pair of adult medaka fish. Here we showed that the distance between the two fish was not maintained in a static condition (Fig. S3). This finding is also consistent with a previous report that the degree of mutual attraction in medaka fish increases according to group size [14].

In many animal groups, certain individuals are consistently observed at the forefront of collective movements, and these individuals have been described as “leaders” [3],[18]. Recently, Harcourt et al. (2009) demonstrated in pairs of sticklebacks that leadership arises from individual differences in the way that fish respond to their partner's movements [18]. Although we could not find an apparent leader in the cohesive movement in the present study, our experimental system will enable investigators to analyze the physical factors and personality characteristics that affect leadership in collective movements.

Conspecific interaction in fish has been shown in many studies using a two-way choice test in which test-fish are given the choice between two stimulus fish or shoals presented behind a transparent wall at either end of a test tank [3],[14],[19]. The association preference of the test fish can be measured by recording the time each fish spends within a certain distance of each stimulus. Some studies have investigated the preference for stimulus fish based on body length and body color. In some fish, including killifish (*Fundulus diaphanus*), association preferences for size-matched fish occur both when shoal-mates are conspecific and when they are heterospecific [20],[21]. In zebrafish, individuals discriminate between shoals having a different pigment pattern and their early experience affects shoaling preference [22]. The cohesive movement in a pair of medaka fish elicited by the OMR was detected in homospecific pairs irrespective of body-color, sex, or albino mutation, but was not detected between heterospecific pairs (medaka-pufferfish, and medaka-zebrafish). Considering that the body length of pufferfish (3 cm) is similar to that of medaka, conspecific interaction in the cohesive movement of the two fish may be mediated by factors other than body-color or body-length, such as movement pattern or morphology.

We also demonstrated that the ability of the schooling-like movement develops during post-hatch growth. In silverside (*Menidia menidia*) [12], and minnow (*Phoxinus phoxinus*) [23], shoaling is not observed just after hatching and physiologic mechanisms for shoaling are formed during post hatching development. Interestingly, Masuda and Tsukamoto (1998) also analyzed the schooling capacity and the OMR using striped jack (*Pseudocaranx dentex*) juveniles and demonstrated that there is a long time-lag between the onset of the OMR (body length 4–6 mm) and that of schooling (body length 16>mm) [24]. Further, Shaw and Tucker noted common properties of fish movement between fish schooling and the OMR [13] and proposed that schooling is based on two orders in the brain: parallel orientation in schooling and the OMR on the lower order, and mutual attraction on a higher order [4]. Development during the juvenile stage might contribute to the emergence of physiologic mechanisms for mutual attraction (higher order) in some fish. As medaka visual function (such as spatial and temporal resolution) improves during growth [25], the ontogenetic improvement of visual function may be necessary for the perception and recognition of conspecific fish.

There are many studies on ‘free swimming’ fish grouping behavior [9],[26]–[29]. Furthermore, coordinated movement between pairs of fish in a static environment has been demonstrated in the bigeye scad *Selar crumenophthalmus* and the barred flagtail *Kuhlia mugil* [30]. An experimental system using zebrafish, an organism in which molecular genetics studies can feasibly be performed, has also been used to demonstrate movement of pairs of fish [31]. In these studies, however, the coordinated and cohesive movement was investigated under a static condition. Here we demonstrated that schooling-like movement in a pair of fish (coordinated and cohesive movement) can be induced by eliciting a simultaneous OMR and that the movement is not observed under a static condition. This is the first evidence that the OMR is a trigger stimulus for cohesive and coordinated movement in a pair of fish. This system enables us to investigate proximate causations of coordination between animals under a dynamic condition. Considering that medaka fish are used extensively in molecular biology and genetic studies [32]–[36], this experimental system will help to clarify the molecular/neural mechanisms underlying the induction of coordinated and cohesive movement between fish.

## Materials and Methods

### Animals

Medaka (*Oryzias latipes*) of drR strain, which contain a red pigment color marker on Y chromosome, (more than 3 months after hatching), Quintet mutant strain (more than 3 months after hatching), T5 strain (body-color mutant, more than 3 months after hatching), and wild-type zebrafish (*Danio rerio*) TL2E strain, more than 7 months after hatching) were kept in groups in plastic aquaria (12×13×19 cm). The medaka T5 strain is derived from a southern Japanese population wild-type medaka [37]. The Quintet mutant strain was obtained from A. Shimada [37]. All groups were maintained at a temperature of 25°C and under a 14 h∶10 h light:dark cycle. Dwarf puffers (*Carinotetraodon travancoricus*) were obtained from a local pet shop and housed in a plastic tank (12×13×19 cm). The tank was maintained at a temperature of 28°C and under a 14 h∶10 h light:dark cycle. For behavioral studies using medaka larvae (T5 strain), eggs were placed separately in a 24-well microplate (IWAKI Glass Co, Ltd., Chiba, Japan). The walls of the wells were covered with white opaque paper to avoid interindividual visual interaction after hatching. Within 24 hours after hatching, for the “isolation” test each larval medaka was transferred to a 6-well plate (IWAKI Glass Co, Ltd., Chiba, Japan) with white opaque walls. For the “group” test, four larval medaka were transferred to a 7-cm diameter glass dish. The 6-well plate and glass dish were maintained at a temperature of 28°C and under a 14 h∶10 h light:dark cycle until the behavioral testing was performed.

### The OMR apparatus

The setup used to measure the optomotor response is shown in Fig. 1A. During the experiment, Adult fish (medaka, dwarf puffers and zebrafish) were transferred into the circular transparent Plexiglass tank (15cm diameter, 9cm height) (Fig. 1A). Water depth was about 3–4 cm. The tank was fixed on the pedestal and concentrically surrounded by a striped cylinder (20 cm diameter, 21 cm height). Vertical stripes of black and white (15 bars of the same width, respectively) were printed on a white paper, which was set on the inner surface of the acryl cylinder. The striped cylinder was positioned on a rotatable metal disk which was driven by a motor, IHT6P3 (SERVO, Kiryu, Japan), adjustable to either direction and various speeds using a motor driver, C-30PN (SERVO). In all the experiments, the striped cylinder was rotated in a counterclockwise direction. The fish behavior was monitored from above by a CCD camera, XC-ST70 (SONY, Tokyo, Japan) and recorded on the hard-disk drive of a personal computer (30 frames per second). A series of frames were analyzed using the software Move-tr/2D 7.0 (Library, Tokyo, Japan).The size of apparatus for larval fish was smaller than that for adult fish. The diameter of striped cylinder and tank were 5.7 cm and 4.3 cm, respectively.

### Behavior test procedure

A pair of fish (Fish A and B) was tested in each experiment (Fig. 1B). At the beginning of an experimental session, Fish A was transferred into the circular test tank surrounded by the stationary, striped cylinder, and adapted to the apparatus for approximately 1 min. At the beginning of the cylinder rotation, we confirmed that the fish in the test tank were exhibiting an OMR (following the movement of the striped cylinder) to avoid recording startle behavior, which many of the fish exhibited. After this delay, the OMR of the single fish was recorded for 70 seconds. Two different velocities – approximately 60 and 80 rpm – were tested. The OMR of the single fish was referred to as the “single OMR” test of Fish A. Next, Fish B was tested using the same procedure as for Fish A (“single OMR” test of Fish B). Finally, Fish A and B were placed in the test tank together, surrounded by the stationary cylinder for adaptation for at least 5 min. The striped cylinder was rotated for at least 1 min to elicit a simultaneous OMR in Fish A and B (Fig. 1B). At the beginning of the rotation, we confirmed that both of the fish are moving in a counterclockwise direction to follow the stripes, and then started recording the OMR (“simultaneous OMR” test of the pair). Movement of each fish was recorded for 60 seconds. Using all the movies, the X-Y coordinates and velocities of the fish were detected automatically every 1/30 second (1 frame) using Move-tr/2D 7.0 software (Library, Tokyo). Using medaka and pufferfish, we confirmed that the fish exhibited a high OMR activity (Table S1). As the OMR abilities of zebrafish and larval medaka were low, we analyzed behavioral data of all the fish including those exhibiting low or very weak OMR activity (Table S1).

### Measurement of the distance between two fish

The distance between two individuals in the “simultaneous OMR” experiment of each pair was measured in each frame (Fig. 1B lower panel). To estimate the imaginary distance between two fish with an independent OMR, data from a “single OMR” test of Fish A and B was superimposed and the “imaginary distance” between the two fish was calculated every 1/30 second (Fig. 1B upper panel). To normalize the superimposed control data, 60-second movies were cut out from 70-second recorded movies to set the initial positions of Fish A and B, so that difference in the angles of Fish A and B, which were represented in polar coordinates, was less than 2°.

### Analysis of the relative position of the two fish

We defined the position of Fish A as at a 0° angle in the angle coordinate with the origin at the center of the tank and calculated the angle of the position of Fish B (Fig. 3A). We defined two conditions based on the relative position of the two fish: (1) Fish B seems to go ahead of Fish A (+180° > the angle of fish B >0°) and (2) Fish A seems to go ahead of Fish B (−180° degree < the angle of Fish B <0°). We counted the number of changes in the condition to analyze the relative position of the two fish.

## Supporting Information

Figure S1 Distance between two adult conspecific fish exhibiting a simultaneous OMR. (A) Frequency histogram of the distance between two adult female medaka exhibiting an OMR, showing the integration of all the pairs. (n = 10) (B) Adult puffer pairs exhibiting an OMR. (n = 10) (C) Adult male zebrafish pairs. (n = 13) (D) Adult medaka pairs with different body colors (T5 strain and albino mutant [Quintet]). (n = 10) (E) Other adult medaka pairs of different body colors (n = 14) (male T5 and orange-red strains [male drR]). Error bars indicate standard deviation (SD).
(1.52 MB TIF)

Figure S2 Distance between two conspecific fish exhibiting OMR. (A) Frequency histogram of distance between two adult female medaka exhibiting OMR when the rotation speed of stripes was 80 rpm (n = 10). (B) Frequency histogram of distance between two adult male medaka exhibiting OMR when the rotation speed of stripes was 60 rpm (left) and 80 rpm (right) (n = 9 and 10, respectively). (C) Frequency histogram of distance between adult male and female medaka exhibiting OMR when the rotation speed of stripes was 60 rpm (left) and 80 rpm (right) (n = 12). (D) Frequency histogram of distance between adult male zebrafish when the rotation speed of stripes was 80 rpm (n = 13). Error bars indicate standard deviation (SD).
(1.59 MB TIF)

Figure S3 Induction of coordinated movement by the OMR. (A) Six examples of a temporal change in the distance of the two adult female medaka without an OMR (under a static condition). (B) Frequency histogram of the distance between two adult female medaka without an OMR (under a static condition). Error bars indicate standard deviation (SD). (C) Frequency histogram of the distance between two adult female medaka exhibiting an OMR, before the single OMR tests.
(1.38 MB TIF)

Figure S4 Distance between two medaka larvae exhibiting an OMR. The size of the apparatus for the larval fish was smaller than that for the adult fish. The rotation speed of the stripes was 40 rpm. (A) Frequency histogram of the distance between two larvae reared in isolation for 5 days (left), 7 days (middle), and 10 days (right) after hatching (n = 10). (B) Two larvae reared in a group for 5 days (left), 7 days (middle), and 10 days (right) after hatching (n = 10). Error bars indicate standard deviation (SD).
(1.22 MB TIF)

Figure S5 Distance between two adult xenogeneic fish exhibiting a simultaneous OMR. (A) Frequency histogram of the distance between adult medaka and puffer (n = 10). (B) Frequency histogram of the distance between adult medaka and zebrafish (n = 10). Error bars indicate standard deviation (SD). (C) Distance between medaka and zebrafish when the rotation speed of stripes was 80 rpm. (n = 10). Error bars indicate standard deviation (SD).
(0.87 MB TIF)

Table S1 Summary of the OMR tests.
(0.04 MB DOC)

Movie S1 Two adult conspecific fish (Medaka) exhibiting a simultaneous OMR. Blue line indicates distance (d) between the two fish.
(3.72 MB MOV)

Movie S2 Two adult conspecific fish (pufferfish) exhibiting a simultaneous OMR. Blue line indicates distance (d) between the two fish.
(2.63 MB MOV)

Movie S3 Two adult xenogeneic fish (Medaka and pufferfish) exhibiting a simultaneous OMR. Blue line indicates distance (d) between the two fish.
(3.80 MB MOV)

Movie S4 Two adult conspecific fish (Medaka) exhibiting a simultaneous OMR. An example of numerous changes in the relative position of a pair of fish.
(1.09 MB MOV)

Movie S5 Two adult larval Medaka fish (Isolation 7pdf) exhibiting a simultaneous OMR.
(6.19 MB MOV)
