# Supplementary figures and images for: Coordinated and Cohesive Movement of Two Small Conspecific Fish Induced by Eliciting a Simultaneous Optomotor Response
Source: PLoS One. 2010 Jun 22;5(6):e11248. doi: 10.1371/journal.pone.0011248 (PMC2889830; doi:10.1371/journal.pone.0011248)

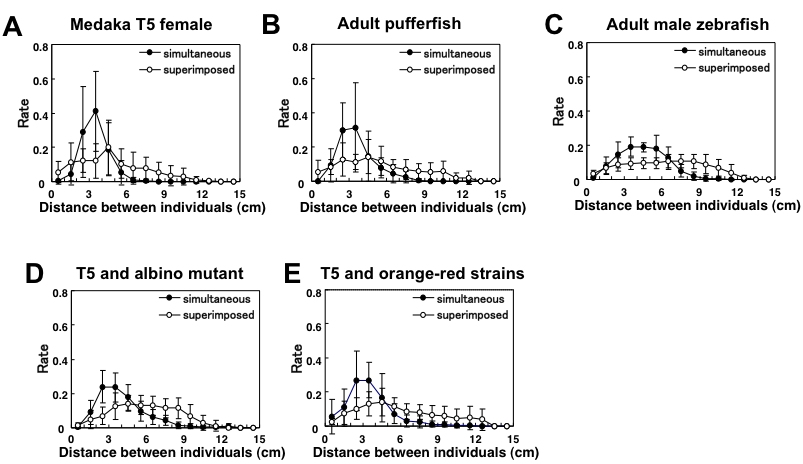

Supplement: Figure S1 — Distance between two adult conspecific fish exhibiting a simultaneous OMR. (A) Frequency histogram of the distance between two adult female medaka exhibiting an OMR, showing the integration of all the pairs. (n = 10) (B) Adult puffer pairs exhibiting an OMR. (n = 10) (C) Adult male zebrafish pairs. (n = 13) (D) Adult medaka pairs with different body colors (T5 strain and albino mutant [Quintet]). (n = 10) (E) Other adult medaka pairs of different body colors (n = 14) (male T5 and orange-red strains [male drR]). Error bars indicate standard deviation (SD). (1.52 MB TIF) [file pone.0011248.s001.tif]

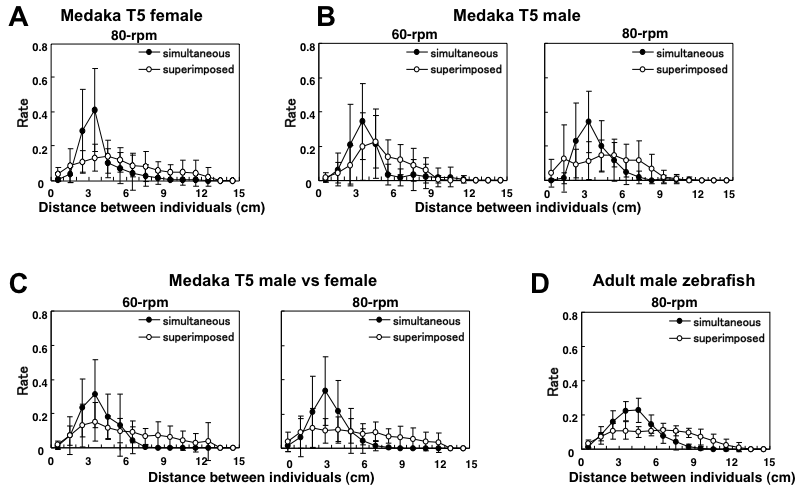

Supplement: Figure S2 — Distance between two conspecific fish exhibiting OMR. (A) Frequency histogram of distance between two adult female medaka exhibiting OMR when the rotation speed of stripes was 80 rpm (n = 10). (B) Frequency histogram of distance between two adult male medaka exhibiting OMR when the rotation speed of stripes was 60 rpm (left) and 80 rpm (right) (n = 9 and 10, respectively). (C) Frequency histogram of distance between adult male and female medaka exhibiting OMR when the rotation speed of stripes was 60 rpm (left) and 80 rpm (right) (n = 12). (D) Frequency histogram of distance between adult male zebrafish when the rotation speed of stripes was 80 rpm (n = 13). Error bars indicate standard deviation (SD). (1.59 MB TIF) [file pone.0011248.s002.tif]

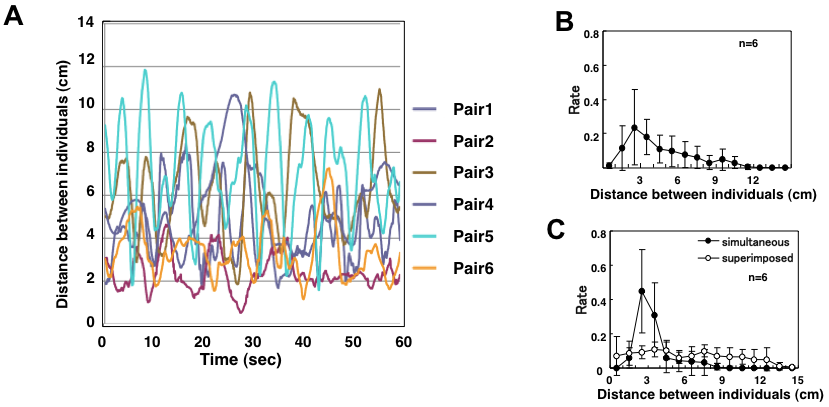

Supplement: Figure S3 — Induction of coordinated movement by the OMR. (A) Six examples of a temporal change in the distance of the two adult female medaka without an OMR (under a static condition). (B) Frequency histogram of the distance between two adult female medaka without an OMR (under a static condition). Error bars indicate standard deviation (SD). (C) Frequency histogram of the distance between two adult female medaka exhibiting an OMR, before the single OMR tests. (1.38 MB TIF) [file pone.0011248.s003.tif]

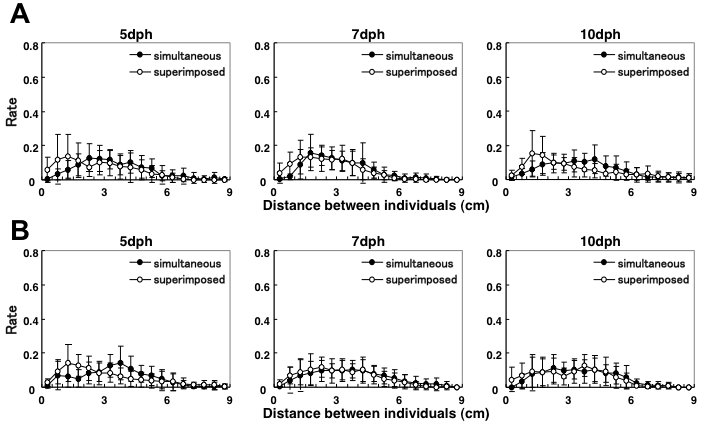

Supplement: Figure S4 — Distance between two medaka larvae exhibiting an OMR. The size of the apparatus for the larval fish was smaller than that for the adult fish. The rotation speed of the stripes was 40 rpm. (A) Frequency histogram of the distance between two larvae reared in isolation for 5 days (left), 7 days (middle), and 10 days (right) after hatching (n = 10). (B) Two larvae reared in a group for 5 days (left), 7 days (middle), and 10 days (right) after hatching (n = 10). Error bars indicate standard deviation (SD). (1.22 MB TIF) [file pone.0011248.s004.tif]

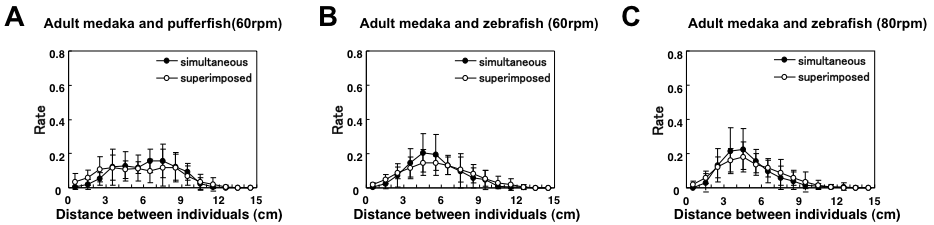

Supplement: Figure S5 — Distance between two adult xenogeneic fish exhibiting a simultaneous OMR. (A) Frequency histogram of the distance between adult medaka and puffer (n = 10). (B) Frequency histogram of the distance between adult medaka and zebrafish (n = 10). Error bars indicate standard deviation (SD). (C) Distance between medaka and zebrafish when the rotation speed of stripes was 80 rpm. (n = 10). Error bars indicate standard deviation (SD). (0.87 MB TIF) [file pone.0011248.s005.tif]
