# Supplementary material for: Coordinated and Cohesive Movement of Two Small Conspecific Fish Induced by Eliciting a Simultaneous Optomotor Response
Source: PLoS One. 2010 Jun 22;5(6):e11248. doi: 10.1371/journal.pone.0011248 (PMC2889830; doi:10.1371/journal.pone.0011248)
Supplement: Table S1 — Summary of the OMR tests. (0.04 MB DOC) [file pone.0011248.s006.doc]

T5 Pufferfish Zebrafish Pufferfish vs T5 Zebrafish vs T5 Quintet vs T5 drR vs T5

Pair1 +++ +++ - +++ + - +++

Pair2 +++ +++ - +++ - - +++

Pair3 +++ +++ + +++ + - +++

Pair4 +++ +++ - +++ + - +++

Pair5 +++ +++ - +++ +++ - +++

Pair6 +++ +++ - +++ - - +++

Pair7 +++ +++ +++ +++ +++ + +++

Pair8 +++ +++ + +++ + - +++

Pair9 +++ +++ + +++ - + +++

Pair10 +++ +++ - +++ +++ +++ +++

Pair11 - +++

Pair12 - +++

Pair13 - +++

Pair14 +++

5pdf-I 5pdf-G 7pdf-I 7pdf-G 10pdf-I 10pdf-G

Pair1 +++ + +++ + +++ +++

Pair2 +++ +++ +++ + +++ +

Pair3 + +++ +++ +++ + +++

Pair4 + +++ +++ +++ + +

Pair5 +++ +++ +++ +++ +++ +++

Pair6 + +++ +++ +++ + +

Pair7 - + +++ +++ +++ +

Pair8 +++ +++ +++ + - +++

Pair9 - + +++ +++ + +++

Pair10 + +++ +++ +++ +++ +++

*OMR test

+++: High OMR activity (R1a, R1b, R2a and R2b> 0.95)

+: Low OMR activity (R1a, R1b, R2a, and R2b ≥0.70)

-: No or week OMR activity (R1a, R1b, R2a, or R2b ≤0.70)

To assess the degree of OMR abilities, we measured the cumulative angle of fish rotation and that of cylinder rotation and calculated the correlation coefficient. First, for “the single OMR”, cumulative angles (ω1A and ω1B) of the two fish (Fish-A, and -B) from their own start point were calculated per 1/30 second (1 frame). The cumulative angle of one stripe in each experiment was measured (ω1Sa, and ω1Sb). Second, the cumulative angle of fish and stripe for “simultaneous OMR” were also calculated (ω2A, ω2b and ω2S). Then, the correlation coefficient (R1a, R1b, R2a and R2b) between ω1A and ω1Sa, ω1B and ω1Sb, ω2A and ω2S and between ω2B and ω2S were calculated in each experiment.
